# *Alu*-Mediated *MEN1* Gene Deletion and Loss of Heterozygosity in a Patient with Multiple Endocrine Neoplasia Type 1

**DOI:** 10.1210/jendso/bvaa051

**Published:** 2020-05-09

**Authors:** Satoshi Yoshiji, Yorihiro Iwasaki, Kanako Iwasaki, Sachiko Honjo, Koichi Hirano, Katsuhiko Ono, Yuto Yamazaki, Hironobu Sasano, Akihiro Hamasaki

**Affiliations:** 1 Department of Diabetes and Endocrinology, Tazuke Kofukai Medical Research Institute, Kitano Hospital, Osaka, Japan; 2 Department of Laboratory Medicine, Tazuke Kofukai Medical Research Institute, Kitano Hospital, Osaka, Japan; 3 Department of Anatomic Pathology, Tohoku University Graduate School of Medicine, Miyagi, Japan

**Keywords:** multiple endocrine neoplasia type 1, *Alu* retrotransposon, *Alu*/*Alu*-mediated genomic rearrangement, loss of heterozygosity

## Abstract

Multiple endocrine neoplasia type 1 (MEN1) is an autosomal dominant disorder caused by mutations of the tumor suppressor gene *MEN1*. Most of the germline *MEN1* gene mutations have been small mutations, and the whole gene deletion is rarely observed. In the present study, we revealed *Alu* retrotransposon-mediated *de novo* germline deletion of the whole *MEN1* gene and somatic copy-neutral loss of heterozygosity (LOH) in a patient with MEN1. The patient is a 39-year-old woman who was referred to our department for the management of prolactinoma. She was also diagnosed with primary hyperparathyroidism and suspected of MEN1. Although nucleotide sequencing did not detect any *MEN1* gene mutations, multiplex ligation-dependent probe amplification (MLPA) revealed a large germline deletion of the *MEN1* gene. Subsequent quantitative polymerase chain reaction (qPCR)–based copy number mapping showed a monoallelic loss of approximately 18.5-kilobase region containing the whole *MEN1* gene. Intriguingly, the 2 breakpoints were flanked by *Alu* repetitive elements, suggesting the contribution of *Alu*/*Alu*-mediated rearrangements (AAMR) to the whole *MEN1* gene deletion. Furthermore, copy number mapping using MLPA and qPCR in combination with single nucleotide polymorphism analysis revealed copy-neutral LOH as a somatic event for parathyroid tumorigenesis. In conclusion, copy number mapping revealed a novel combination of *Alu*/*Alu*-mediated *de novo* germline deletion of the *MEN1* gene and somatic copy-neutral LOH as a cytogenetic basis for the MEN1 pathogenesis. Moreover, subsequent in silico analysis highlighted the possible predisposition of the *MEN1* gene to *Alu* retrotransposon-mediated genomic deletion.

Multiple endocrine neoplasia type 1 (MEN1) is an autosomal dominant disorder caused by mutations of the tumor suppressor gene *MEN1*. Tumorigenesis in MEN1 follows biallelic inactivation of the tumor suppressor gene *MEN1*, consistent with Knudson’s two-hit hypothesis [1]. Although the majority of MEN1 cases are caused by point mutations, there are a few cases with no detectable defects by Sanger sequencing [2], in which copy number analysis may be required to identify a large deletion [3]. However, little is known about the risk for chromosomal deletions in the *MEN1* gene locus. Moreover, as for the somatic event in MEN1-associated tumors, while the loss of heterogeneity (LOH) is frequently observed at 11q13 [4, 5], somatic copy number alteration has not been characterized.

In the present study, we revealed *Alu* retrotransposon-mediated *de novo* germline deletion of the whole *MEN1* gene (“first hit”) in combination with somatic copy-neutral loss of heterozygosity (LOH) (“second hit”) as the cytogenetic basis for the MEN1 pathogenesis.

## Patient and Methods

### Case description

**Figure 1.**
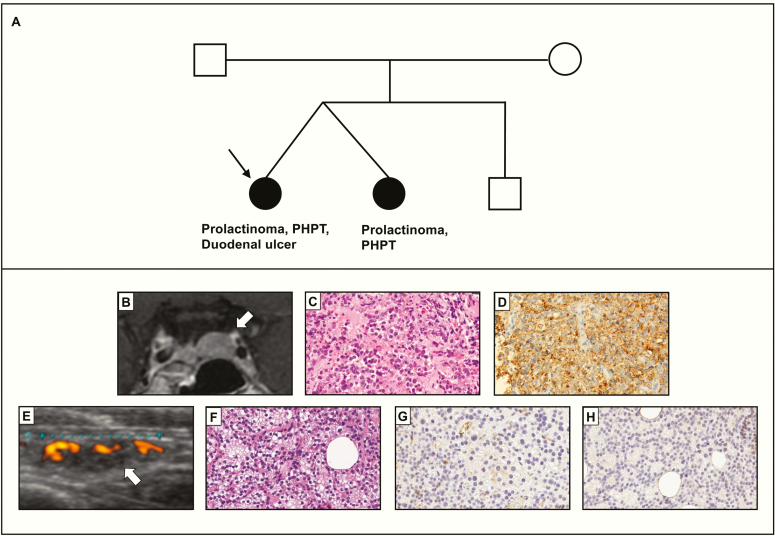
Clinical characteristics of the proband. (A) The family pedigree of the proband (arrow). (B) T1-weighted MRI image of the pituitary tumor. (C, D) Hematoxylin and eosin staining (C) and immunostaining for prolactin (D) of the pituitary tumor. (E) Ultrasound image of the right upper parathyroid gland. (F) Hematoxylin and eosin staining of the hyperplastic right upper parathyroid. (G, H) Menin immunostaining of the right upper (G) and left lower (H) parathyroid. Abbreviation: PHP, primary hyperparathyroidism.

A 39-year-old woman was referred to the endocrinology clinic for the management of prolactinoma (241.5 μg/L at presentation; normal range, 3.7–16.3). She suffered from amenorrhea since age 32 and had a past medical history of multiple bone fractures, urolithiasis, and gastroduodenal ulcer. Her family history was remarkable for prolactinoma and primary hyperparathyroidism of her twin sister (Fig. 1A). She underwent transsphenoidal surgery at our institution for cabergoline-resistant prolactinoma. Pathological diagnosis was prolactinoma (Fig. 1B, 1C and 1D). Her prolactin level was normalized postoperatively, and menstruation was restored. Apart from prolactinoma, she had hypercalcemia (2.8 mmol/L; normal range, 2.2–2.5) and elevated serum intact parathyroid hormone level (33.6 pmol/L; normal range, 2.0–9.3). Both right upper and left lower parathyroid glands were enlarged on ultrasound (Fig. 1E). She underwent resection of all four parathyroid glands, and pathological diagnosis was parathyroid hyperplasia (Fig. 1F, 1G and 1H). Clinical diagnosis of MEN1 was made and genetic analysis was performed.

## Methods

### Genetic analysis

Deoxyribonucleic acid (DNA) was extracted from peripheral leukocytes and resected parathyroid tissues (right upper and left lower glands) using QIAamp DNA Mini Kit (Qiagen, Hilden, Germany). MLPA was performed with SALSA P017 MEN1 kit (MRC Holland, Amsterdam, the Netherlands). qPCR was performed with THUNDERBIRD SYBR qPCR Mix (Toyobo, Osaka, Japan) using StepOnePlus (Applied Biosystems, Foster City, CA, US). A total of 11 primers sets (p1-11) were used for the analysis of germline and somatic mutations. Primers p1-3 were designed to target loci upstream of the *MEN1* gene; p4-5, those within the *MEN1* gene; and p6–11 those downstream of the *MEN1* gene. Primer sequences are shown in Table 1. Relative copy number was calculated by the ΔΔCT method using the amplicon p10 as the reference locus. End-point polymerase chain reaction (PCR) was performed using KOD One (Toyobo, Osaka, Japan). Sanger sequencing was performed with 3730xl DNA analyzer (Applied Biosystems, Foster City, CA, US).

**Table 1.** Primer sequences.

Primers	Sequence (5’->3’)
p1	
Forward	CTTCACTACCTTCTCCAGACAGTTC
Reverse	AGGAGGGAGCAGAATGTCTATAAGT
p2	
Forward	GTGATTTGAAGTAGAATGGTCAGG
Reverse	GGGCATATGTGGTGGGTAATTAG
p3	
Forward	CTCAAAGTCGCATACTCCCGAG
Reverse	TCATTGCAGATGAGAGACCAAGG
p4	
Forward	GCTGGCTGTACCTGAAAGGATCA
Reverse	CGAGTCGGTGTGCAGGTCAATG
p5	
Forward	GCTGGGTCCTAATTACCAGTCTT
Reverse	ATATACTCCTAGGGGCTGAGTGG
p6	
Forward	ACTGAGAGATAAGACTCGCTGGTAA
Reverse	CTGACTCAGATGGTCTGTAGTAGCC
p7	
Forward	CTACACACTCAACCTGCATGAACT
Reverse	ACCTTGTACCTGAGAGTGACAGC
p8	
Forward	TTACTGAGCACTTATGCTATGTTGG
Reverse	GGATTACAGGATTGGGATTACAGG
p9	
Forward	GAAAGTGTGTCAGGGTTTCTAGGC
Reverse	CAGGCAAACTCTTAACAGCTCCC
p10	
Forward	TCTTTTGCAAGTTGAGCCAGTA
Reverse	AGGTCCCACTTGCACATCTAAT
p11	
Forward	TCTAGAAGATAAGTTCCTGGAAGCA
Reverse	CCTTCTACTTGTCCTCAAGAATGAC

### In silico analysis

The clinical implication of sequences surrounding the breakpoints was analyzed with Repeatmasker (http://www.repeatmasker.org) and UCSC Genome Browser (http://genome.ucsc.edu).

### Immunohistochemistry

Paraffin-embedded tumor tissues of surgically resected parathyroid glands (right upper and left lower glands) and pituitary glands were used for immunohistochemistry analysis. Menin was stained using a rabbit polyclonal anti-menin antibody (Abcam, Cambridge, UK; ab2605, dilution 1:3000). Prolactin was stained using a rabbit polyclonal antiprolactin antibody (Cell Marque, Rocklin, CA; EP193, dilution 1:100).

### Ethics statement

Informed consent was obtained from the patient for the genetic testing and the publication of this article. All clinical investigations and genetic analysis were performed according to the guidelines of the Declaration of Helsinki and approved by the local ethics committee of Kitano Hospital (#180400601).

## Results

### Analysis of germline *MEN1* deletion

**Figure 2.**
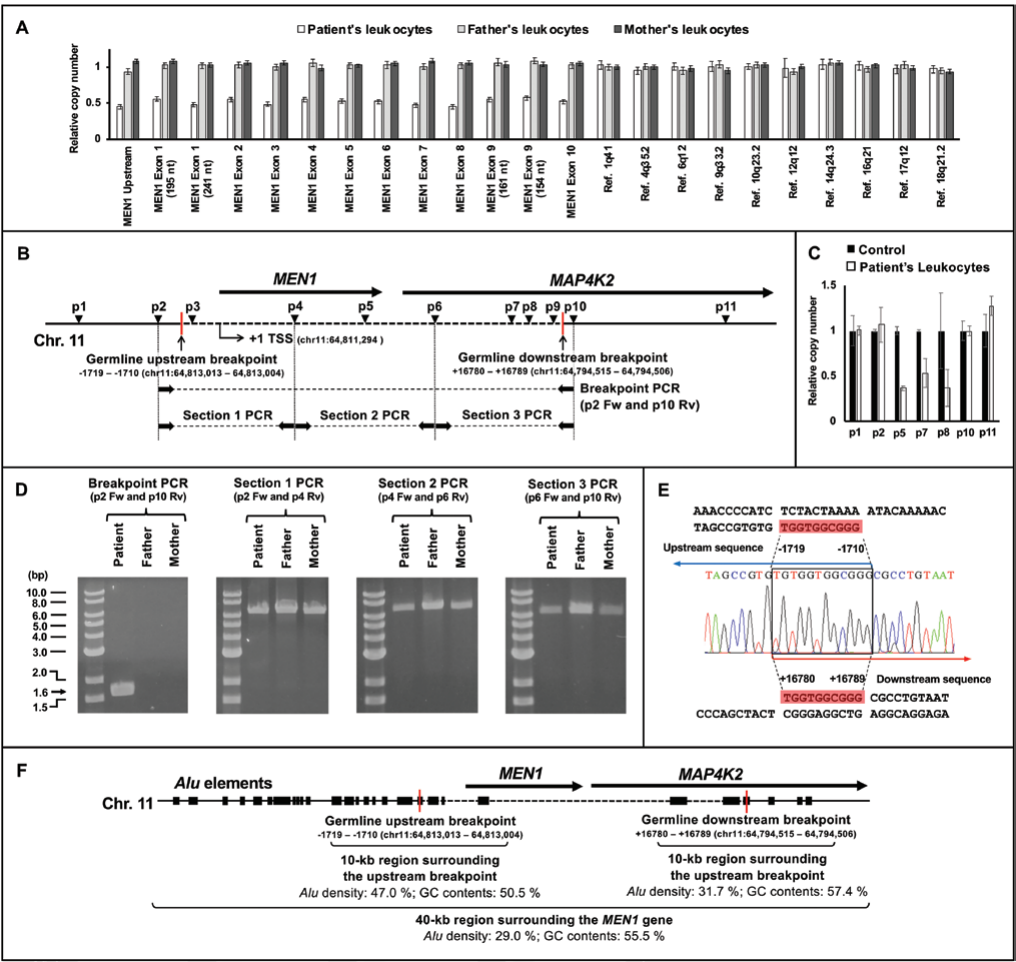
Analysis of germline *MEN1* gene deletion. (A) Multiplex ligation-dependent probe amplification (MLPA) analysis of the *MEN1* gene of leukocytes from the patient and her parents. Ligation sites of MLPA probes include upstream region of the *MEN1* gene, exon 1-10 of the *MEN1* gene, and reference regions (positive controls). (B) Locations of primers (p1-p11) used for genetic analysis of the germline *MEN1* deletion. Primer pairs of p2 forward and p10 reverse were used in polymerase chain reaction (PCR) identifying the germline breakpoints (Breakpoint PCR). Red vertical lines indicate the upstream and downstream breakpoints, whose locations are shown below (where + 1 is the *MEN1* transcription start site). (C) Quantitative PCR–based copy number mapping of the region surrounding the *MEN1* gene. Relative copy number of the region in the control leukocytes of a healthy adult (black box) and the patient’s leukocytes (white box) are shown. (D) Primer pairs of p2 forward (Fw) and p10 reverse (Rv) shown in Fig. 2B were used for PCR identifying the germline breakpoints (Breakpoint PCR). A 1.6-kb patient-specific product generated in the breakpoint PCR is marked with a black arrow. The 20-kb region flanked by primers p2 Fw and p10 Rv was divided to 3 segments (sections 1, 2, and 3) (Fig. 2A) and amplified using primer pairs of p2 Fw and p4 Rv, p4 Fw and p6 Rv, and p6 Fw and p10 Rv, respectively, in the leukocytes of the patient and parents. (E) Chromatogram analysis of the patient-specific product obtained in the breakpoint PCR. (F) Schematic representation of the density of *Alu* elements and guanine-cytosine contents in the 40-kb region surrounding the *MEN1* gene. *Alu* elements are plotted as the black boxes on chromosome 11q13. Red vertical lines indicate the upstream and downstream breakpoints, whose locations are shown below (where +1 is the *MEN1* transcription start site). Locations of the breakpoints in GRCh38.p12 are also provided in parenthesis. The error bars in Fig. 2A and 2C represent standard deviation. Abbreviations: Chr. 11, chromosome 11; Ref, reference; TSS, transcription start site.

Although direct sequencing of the *MEN1* gene in the patient’s leukocytes did not find any germline pathogenic variants of the *MEN1* gene, MLPA revealed that the copy number was reduced to about half in all of the exons, suggesting a large deletion including the whole *MEN1* gene (Fig. 2A and Supplementary Fig. 1 [6]). Notably, MLPA showed no copy number reduction in leukocytes of her parents, suggesting the patient had a *de novoMEN1* germline deletion. Her sister’s genome was not available for the analysis. To narrow the germline breakpoints of the patient, we performed a qPCR-based copy number mapping with primers p1, p2, p5, p7, p8, p10, and p11 (Fig. 2B and 2C). The copy number mapping revealed the copy number was reduced by about half at regions targeted by primers p5, p7, and p8, whereas no copy number reduction was observed at regions targeted by primers p1, p2, p10, and p11 (Fig. 2C). This suggested the upstream breakpoint was located in between the target regions of p2 and p5 and the downstream breakpoint in between those of p8 and p10. PCR using a primer pair of p2 forward (p2 Fw) and p10 reverse (p10 Rv) generated a product of 1,602 bases (about 1.6 kb) length in the patient’s genome, which is smaller than the expected size of the amplicon (about 20 kb). The 1.6-kb product was not obtained from her parents’ genomes (Fig. 2D). In parallel, we divided the region flanked by primers p2 Fw and p10 Rv to 3 segments (sections 1, 2, and 3) (Fig. 2B) and amplified them separately because the region was too large to amplify (Supplementary Fig. 2 [6]). All segments were amplified from the patient’s and her parents’ genomes. These results collectively showed a *de novoMEN1* gene deletion of the patient.

Sequencing of the PCR product, which was aligned against the human reference genome (GRCh38.p12), revealed that upstream breakpoint was located between −1719 to −1710 and downstream breakpoint between +16780 and +16789 (where +1 is the *MEN1* transcription start site, which is located at chr11:64 811 294 in GRCh38.p12), with exactly identical 10 nucleotides around the breakpoints (5’-TGGTGGCGGG-3’) (Fig. 2E). The deletion was about 18.5 kb length and contained the whole *MEN1* gene and a part of *MAP4K2* gene, which is located downstream of the *MEN1* gene (Fig. 2B).

Notably, both breakpoints were located within *Alu*Sx1 elements, which belong to a family of retrotransposons. The sequences of *Alu*Sx1 were in parallel orientation and highly homologous to each other (81.5%). The density of *Alu* elements was higher in the 10.0-kb proximity of upstream and downstream breakpoints (47.0% and 31.7%, respectively) in contrast to a 40.0-kb region surrounding the *MEN1* gene (29.0%). Guanine-cytosine contents were also relatively rich in the 10.0-kb proximity of upstream and downstream breakpoints (50.5% and 57.4%, respectively). (Fig. 2F).

### LOH analysis

**Figure 3.**
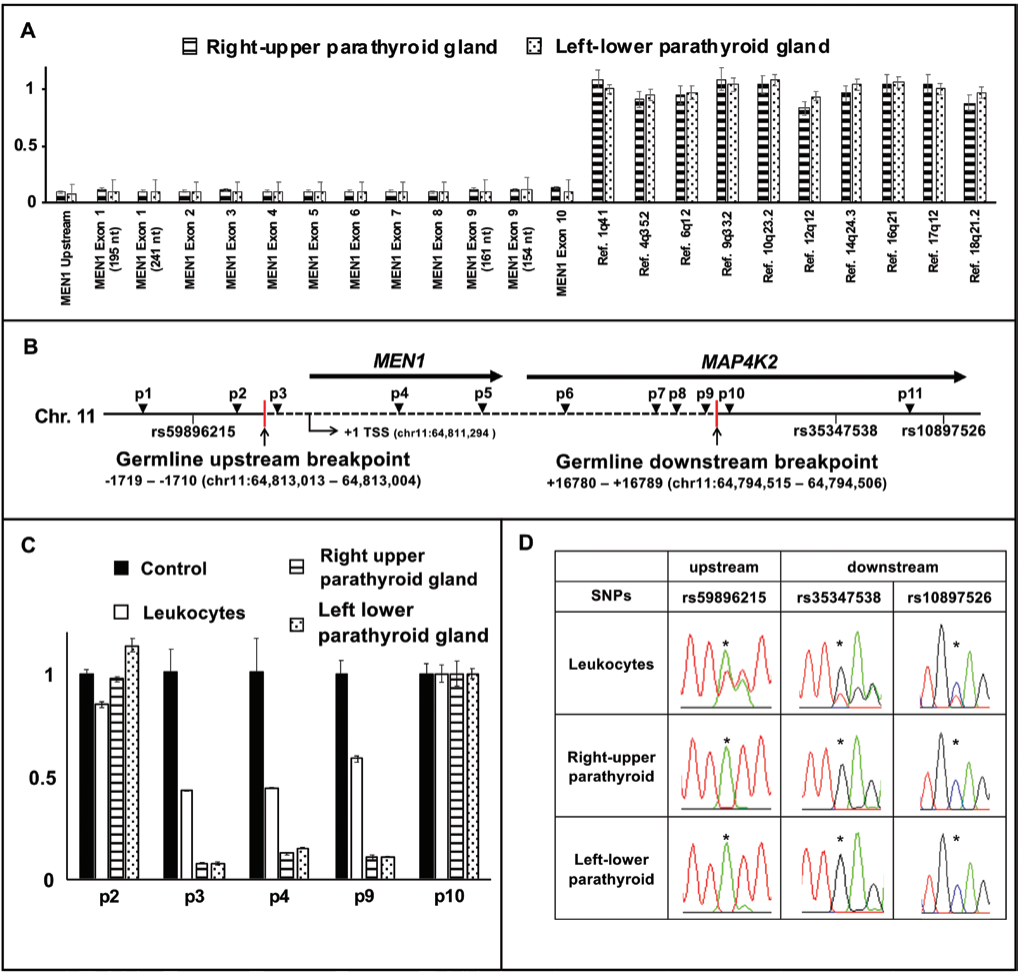
Somatic loss of heterozygosity (LOH) analysis. (A) Multiplex ligation-dependent probe amplification (MLPA) analysis of the *MEN1* gene of right upper and left lower parathyroid glands. Ligation sites of MLPA probes include upstream region of the *MEN1* gene, exon 1-10 of the *MEN1* gene, and reference regions (positive controls). (B) Locations of primers (p1-p11) used for genetic analysis of the germline *MEN1* deletion and somatic LOH. Locations of relevant upstream and downstream single nucleotide polymorphisms (SNPs) are also shown. Red vertical lines indicate the upstream and downstream breakpoints, whose locations are shown below (where +1 is the *MEN1* transcription start site). (C) Quantitative polymerase chain reaction-based copy number mapping of the deleted region. Relative copy number of the region in the control leukocytes of a healthy adult (black box), the patient’s leukocytes (white box), right upper (dashed box), and left lower (dotted box) hyperplastic parathyroid glands of the patient are shown. (D) Analysis of SNPs (rs59896215, rs35347538, and rs10897526) surrounding the breakpoints shown in Fig. 3B. Germline heterozygosity was lost in both right upper and left lower hyperplastic parathyroid glands (marked with an asterisk). The error bars in Fig. 3A and 3C represent standard deviation Abbreviation: Chr. 11, chromosome 11.

To identify the somatic “second hit,” we analyzed genomic DNA of resected parathyroid lesions by MLPA and qPCR. MLPA showed the copy number was reduced by more than 90% in all of the exons of the *MEN1* gene, consistent with a large somatic deletion including the whole *MEN1* gene (Fig. 3A). qPCR-based copy number mapping using the primers flanking the breakpoints: p2, p3, p4, p9, and p10 also showed that the copy number was markedly reduced to almost zero in the same region where germline deletion was found (target region of the primer p3, p4, and p9) (Fig. 3B and 3C). These results were consistent with the presence of somatic LOH. Furthermore, germline heterozygosity for single nucleotide polymorphisms adjacent to the breakpoints was lost in parathyroid lesions (Fig. 3D). Although exact somatic breakpoints were not determined, these findings collectively suggest copy-neutral LOH due to acquired uniparental disomy is the somatic event that led to parathyroid hyperplasia. The loss of menin expression of the patient’s parathyroid glands also supported biallelic inactivation of the *MEN1* (Fig. 1G and 1H).

## Discussion

In the present case, copy number mapping of the genomic copy number showed not only germline retrotransposon-mediated *de novo* MEN1 gene deletion (“first hit”), but also somatic copy neutral-LOH (“second hit”) as the cytogenetic basis for the MEN1 pathogenesis. As for the first hit, it is plausible that a *de novo* germline deletion of the MEN1 gene was mediated by two *Alu*Sx1 repetitive sequences found around both breakpoints. Alu elements are repetitive sequences which number about 1.1 million copies in the human genome [7]. *Alu* sequences are highly homologous to each other, and *Alu* recombination-mediated genomic deletion called AAMR is associated with a number of genetic disorders [8]. In AAMR, a mispairing of 2 similar *Alu* sequences causes unequal nonallelic homologous recombination, which leads to the formation of a *de novo* chimeric *Alu* element and genomic disruption. Although the risk for AAMR of the *MEN1* locus is not yet fully determined, there are several genes that are known to be particularly prone to recurrent AAMR, such as *LDLR* [9]. These high-risk loci have several features: high sequence similarity (70%-100%), parallel orientation, high density of *Alu* elements, and high guanine-cytosine content in the nearby regions [9]. Notably, the surrounding region of the present breakpoints and *Alu*Sx1 elements met all of the previously described features, suggesting the possible predisposition of the *MEN1* gene to the AAMR.

Concerning somatic second hit in *MEN1*, 11q13 LOH is found in almost 100% of MEN1-related parathyroid tumors, whereas 11q13 LOH is found in only 30% to 40% of sporadic parathyroid tumors [4,5,10]. Although there are a few reports on uniparental disomy in sporadic parathyroid carcinoma [11], this is the first report of the copy-neutral LOH in MEN1-related parathyroid hyperplasia.

## Conclusions

The copy number mapping revealed a novel combination of *Alu*/*Alu*-mediated *de novo* germline deletion of the *MEN1* gene and somatic copy-neutral LOH as a cytogenetic basis for the MEN1 pathogenesis, which cannot be detected by Sanger sequencing. Moreover, subsequent in silico analysis highlighted the possible predisposition of the *MEN1* gene to *Alu* retrotransposon-mediated genomic deletion.

## Additional Information

***Disclosure Summary:*** The authors declare no conflicts of interest.

***Data Availability:*** The data sets generated during and analyzed during the current study are not publicly available but are available from the corresponding author on reasonable request.
